# Development and Internal Multicenter Validation of a Deep Learning Model for Predicting Post-Hepatectomy Liver Failure in Patients with Hepatocellular Carcinoma: A Multicenter Study

**DOI:** 10.3390/cancers18060926

**Published:** 2026-03-12

**Authors:** Qian Chen, Feng Xia, Bin Guo, Zhicheng Liu, Xulin Liu, Chang Shu, Jing Yan, Zhancheng Qiu, Qiao Zhang, Zhenheng Wu, Zhiyuan Huang, Xiaoping Chen, Bixiang Zhang, Peng Zhu

**Affiliations:** 1 Department of Hepatobiliary Surgery, The First Affiliated Hospital of Shihezi University, Shihezi 832000, China; chenqian@shzu.edu.cn; 2 Department of Hepatobiliary and Pancreatic Surgery, Tongji Hospital, Tongji Medical College of Huazhong University of Science and Technology, Wuhan 430023, Chinabixiangzhang@hust.edu.cn (B.Z.); 3 Department of Ultrasound in Medicine, The Second Affiliated Hospital of Zhejiang University School of Medicine, Hangzhou 310009, China; 4 Department of General Surgery, West China Hospital, Sichuan University, Chengdu 610041, China; 5 Department of Hepatic Surgery, Zhongshan People’s Hospital Affiliated to Guangdong Medical University, Zhongshan 528403, China; 6 Department of Hepatobiliary Surgery, Fujian Medical University Union Hospital, Fuzhou 350001, China; 7 Department of General Surgery, General Hospital of Central Theater Command, Wuhan 430000, China

**Keywords:** hepatocellular carcinoma, post-hepatectomy liver failure, deep learning, logistic regression, predictive modeling

## Abstract

Background/Objectives: Post-hepatectomy liver failure is one of the most serious complications after liver resection for hepatocellular carcinoma and is associated with high morbidity and mortality. Traditional clinical scoring systems and statistical models provide limited predictive accuracy. This study aimed to develop and internally validate a deep learning model for predicting post-hepatectomy liver failure using multicenter clinical data. Methods: A retrospective cohort of 498 patients from six centers undergoing curative-intent liver resection for hepatocellular carcinoma was analyzed. Preoperative biochemical parameters, intraoperative surgical variables, and tumor-related characteristics were incorporated into a deep neural network, with logistic regression as a baseline comparator. Data splitting was performed before preprocessing, and imputation/scaling parameters were fitted on the training set only to prevent information leakage. Discrimination was assessed using the area under the receiver operating characteristic curve (AUC) and precision–recall (PR) curves; calibration was assessed using calibration plots and Brier score; and clinical utility was assessed using decision curve analysis (DCA). A sensitivity analysis using a preoperative-only feature set (excluding intraoperative variables) was also conducted. SHapley Additive exPlanations were used to determine variable importance. Results: The deep learning model achieved AUCs of 0.914, 0.892, and 0.906 in the training, validation, and test sets, outperforming logistic regression (0.782, 0.757, and 0.773). Key predictors included ALBI and MELD scores, prothrombin time, intraoperative blood loss, and resection extent. Calibration and decision curve analysis further supported the robustness and clinical utility of the model. Conclusions: The deep learning model provides improved predictive performance for post-hepatectomy liver failure compared with logistic regression in an internally validated multicenter cohort and may support perioperative risk stratification and surgical planning.

## Simple Summary

Post-hepatectomy liver failure is a serious complication that can occur after liver surgery for liver cancer and may lead to poor recovery or even death. Predicting which patients are at high risk before surgery remains difficult using traditional clinical tools. In this study, we developed a deep learning model that analyzes many clinical and surgical factors at the same time to provide a more accurate prediction of liver failure after surgery. The model was tested in patients from multiple hospitals and showed strong and consistent performance. By helping surgeons identify high-risk patients earlier, this model may support safer surgical planning and improve postoperative care.

## 1. Introduction

Post-hepatectomy liver failure (PHLF) is a severe and potentially life-threatening complication following liver resection for hepatocellular carcinoma (HCC). It significantly impacts patient prognosis, leading to increased morbidity and mortality [1,2]. PHLF occurs when the remaining liver is unable to meet the metabolic and synthetic demands of the body, resulting in hyperbilirubinemia, coagulopathy, ascites, and multi-organ dysfunction. Despite advancements in surgical techniques and perioperative management, the accurate prediction of PHLF remains a major clinical challenge [3,4,5]. The ability to predict PHLF risk preoperatively (for risk stratification and patient selection) and to update risk perioperatively (as intraoperative information becomes available) is essential for optimizing surgical planning and postoperative care.

Previous studies have attempted to predict PHLF using clinical scoring systems, biochemical markers, and traditional statistical models. For example, the Model for End-Stage Liver Disease (MELD) score and the Albumin–Bilirubin (ALBI) score have been widely used for assessing liver function preoperatively, but their predictive power for PHLF is limited [6,7]. Logistic regression models have also been utilized to identify risk factors for PHLF, such as preoperative bilirubin levels, platelet count, and extent of liver resection [8,9]. However, traditional statistical methods often struggle to capture the complex, non-linear interactions among multiple risk factors [10], leading to suboptimal prediction accuracy.

In recent years, machine learning models, particularly deep learning, have demonstrated superior predictive performance in medical applications due to their ability to analyze large datasets and uncover hidden patterns within clinical variables [11,12,13]. Studies have shown that deep learning models can outperform traditional regression-based approaches in predicting postoperative complications, including acute kidney injury, sepsis, and mortality in surgical patients [14,15]. However, the application of deep learning in predicting PHLF has not been extensively explored.

This study aims to fill this gap by developing and validating a deep learning model for predicting PHLF in HCC patients undergoing liver resection. To assess the model’s predictive performance, we compare it with a logistic regression model, which serves as a baseline. By leveraging a multicenter dataset of 498 patients, this study seeks to establish a robust and clinically applicable prediction tool that can support preoperative decision-making, enhance patient selection, and ultimately improve postoperative outcomes in liver surgery.

## 2. Materials and Methods

### 2.1. Study Design and Data Collection

A retrospective multicenter study was conducted on 498 HCC patients who underwent hepatectomy between January 2018 and December 2020 at six medical centers. This study aimed to develop and validate a deep learning model for predicting PHLF and compare its performance with a logistic regression model. Comprehensive clinical data were collected from electronic medical records, including demographic characteristics, preoperative liver function markers, tumor features, and intraoperative variables. The dataset was de-identified to ensure patient confidentiality. Inclusion criteria included pathologically confirmed HCC, curative-intent liver resection, and availability of preoperative serological and postoperative liver function data. Exclusion criteria included patients with pre-existing chronic liver failure or decompensated cirrhosis, liver transplantation instead of partial hepatectomy, and missing critical clinical or follow-up data. This study was approved by the Institutional Review Board of all participating institutions and conducted in accordance with the Declaration of Helsinki. Major hepatectomy was defined as resection of three or more Couinaud liver segments. Because patients from all participating centers were pooled and split at the patient level, model evaluation represents internal multicenter validation.

### 2.2. Outcome Definition and PHLF Monitoring

Post-hepatectomy liver failure was defined according to the International Study Group of Liver Surgery (ISGLS) criteria [16], which categorizes PHLF as an impairment in the liver’s ability to maintain its synthetic, excretory, and detoxification functions following hepatic resection. PHLF is characterized by prolonged coagulopathy and hyperbilirubinemia on or after postoperative day five, with no other identifiable causes such as bile leakage or sepsis.

PHLF severity is graded as follows: Grade A: No specific clinical management is required, and the liver dysfunction resolves without intervention; Grade B: Patients require medical intervention, such as fluid management, diuretics, or albumin supplementation, but do not need invasive support; Grade C: Patients require intensive care support, including vasopressors, dialysis, or mechanical ventilation [17]. For this study, patients classified as Grade B or C PHLF were considered positive cases for model training, and the Grade B/C distribution is reported in Table 1.

Postoperative liver function was assessed through daily blood tests during hospitalization and subsequent weekly outpatient follow-ups for up to three months. Parameters monitored included total bilirubin, prothrombin time (international normalized ratio), albumin, ammonia levels, and clinical signs of hepatic decompensation such as ascites and encephalopathy.

### 2.3. Data Preprocessing

To ensure optimal model performance and reduce bias, we applied a split-first preprocessing workflow to prevent information leakage. After stratified splitting, imputation and scaling parameters were fitted on the training cohort only and then applied to the validation and test cohorts. Continuous variables were standardized to z-scores (mean 0, SD 1) based on the training set statistics. Missing values were low (all variables < 10% missingness) and were imputed using the training-set mean for continuous variables and the training-set mode for categorical variables (Supplementary Table S1). Feature selection (univariate screening plus clinical expert review) was performed within the training cohort only to avoid optimistic bias.

Stratified random sampling was used to divide the pooled dataset into training (70%), validation (15%), and test (15%) sets, preserving the incidence of PHLF across splits. The training set was used for model development, the validation set for hyperparameter tuning and threshold selection, and the test set was reserved for final evaluation on unseen data. Because splitting was performed at the patient level rather than by holding out entire centers, this evaluation constitutes internal multicenter validation.

### 2.4. Model Development

Two models were constructed to evaluate the accuracy of PHLF prediction. For a fair comparison, the logistic regression and deep learning models were trained using the same finalized feature set. The logistic regression model served as the baseline comparator and was built using clinically relevant predictors.

The deep learning model was developed using a fully connected deep neural network architecture. The input layer incorporated the full set of preprocessed clinical variables. The final network comprised three hidden layers (108, 72, and 48 neurons) with ReLU activation and dropout regularization (dropout rate = 0.2). The output layer used a sigmoid activation function to predict the probability of PHLF. The model was optimized with the Adam optimizer (learning rate = 0.001) using binary cross-entropy loss, and early stopping was used to reduce overfitting [18]. Hyperparameter tuning was conducted on the training/validation cohorts using grid search (search space and final selected values are reported in Supplementary Table S2). To address class imbalance, class weights inversely proportional to class frequencies were applied during training, and the classification threshold was selected on the validation cohort [19]. The total number of trainable parameters is reported in Supplementary Table S6, and learning curves are provided in Supplementary Figure S4.

### 2.5. Model Evaluation

To assess model performance, we evaluated discrimination, calibration, and clinical utility. Discrimination was assessed using AUC, accuracy, sensitivity, specificity, precision, and F1-score, and additionally summarized using precision–recall (PR) curves. Calibration was assessed using calibration plots and Brier score (with lower values indicating better calibration). Clinical usefulness was assessed using decision curve analysis (DCA) to quantify net benefit across a range of threshold probabilities.

To enhance interpretability, feature importance analysis was conducted using SHapley Additive exPlanations values to identify key predictive factors in the deep learning model. By systematically developing and validating these models, this study aims to establish an accurate and clinically applicable artificial intelligence-assisted prediction tool for PHLF prediction, ultimately improving surgical risk stratification and postoperative management.

### 2.6. Data Analysis

Continuous variables were presented as median with interquartile range (IQR) or mean ± standard deviation (SD), as appropriate, and categorical variables were expressed as frequencies with percentages. For comparisons across the three cohorts (training/validation/test), categorical variables were compared using the chi-square test or Fisher’s exact test, as appropriate. For continuous variables, normality was assessed using the Shapiro–Wilk test. Normally distributed variables were compared using one-way analysis of variance (ANOVA), while non-normally distributed variables were compared using the Kruskal–Wallis test.

To identify risk factors for post-hepatectomy liver failure, univariate logistic regression analysis was conducted in the training cohort. Variables with a univariate significance level of *p* < 0.05 were further analyzed using multivariate logistic regression. The final predictors were selected through a backward stepwise approach. To detect multicollinearity, the variance inflation factor (VIF) was calculated, and variables with VIF > 5 were excluded from the final model [20]; candidate variables and corresponding VIF values are provided in Supplementary Table S3.

To compare the performance of the logistic regression and deep learning models, receiver operating characteristic (ROC) curves were generated, and AUC was used as the primary performance metric. Feature importance was analyzed for both models. In the logistic regression model, importance was determined using odds ratios (ORs) with 95% confidence intervals (CIs).

To evaluate preoperative applicability, we conducted a sensitivity analysis by reconstructing the model after excluding intraoperative variables (operative time and blood loss), while retaining all remaining predictors. The model was trained and evaluated using the same modeling framework and validation strategy as the primary perioperative model.

Statistical analyses were performed using SPSS 25.0 (IBM Corp, Armonk, NY, USA), with *p*-values < 0.05 considered statistically significant. Python (version 3.8, Python Software Foundation, Wilmington, DE, USA) was employed for model development and evaluation. ROC and PR curves, calibration plots/Brier scores, and DCA were generated using Python and/or R (version 4.1.2, Vienna, Austria). Sample size estimation was conducted using PASS software (version 11.0, NCSS LLC, Kaysville, UT, USA); assumptions and results are reported in the Supplementary Text S1.

## 3. Results

### 3.1. Baseline Characteristics of the Training, Validation, and Test Cohorts

**Figure 1:**
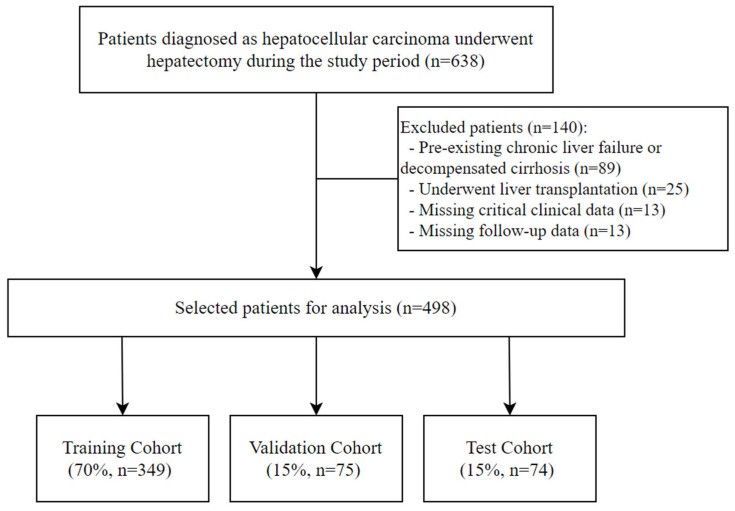
Flow chart for inclusion and exclusion of patients.

A total of 498 HCC patients who underwent liver resection were included. They were randomly assigned to training (70%, n = 349), validation (15%, n = 75), and test (15%, n = 74) cohorts. The inclusion and exclusion process is illustrated in Figure 1. Key clinical characteristics were balanced across cohorts (*p* > 0.05 for all variables), with no significant differences in age, sex, tumor characteristics, or liver function markers, confirming appropriate randomization. Table 1 details the baseline demographic, preoperative, and surgical characteristics of each cohort.

### 3.2. Univariate and Multivariate Logistic Regression Analysis of HCC Patients with PHLF in the Training Cohort

Univariate logistic regression analysis was conducted in the training cohort to assess potential risk factors for PHLF. Variables with *p* < 0.05 in the univariate analysis were included in the multivariate logistic regression model. Multivariate logistic regression identified ALBI score (OR = 1.72, 95% CI: 1.35–2.15, *p* = 0.002), MELD score (OR = 1.55, 95% CI: 1.25–2.00, *p* = 0.008), prothrombin time (OR = 1.78, 95% CI: 1.40–2.22, *p* = 0.001), intraoperative blood loss (OR = 1.38, 95% CI: 1.10–1.70, *p* = 0.015), and extent of resection (major vs. minor, OR = 1.58, 95% CI: 1.25–2.08, *p* = 0.007) as significant independent predictors of PHLF. The full results of univariate and multivariate logistic regression analyses are presented in Table 2.

### 3.3. Survival Analysis Comparing PHLF and Non-PHLF Groups

**Figure 2:**
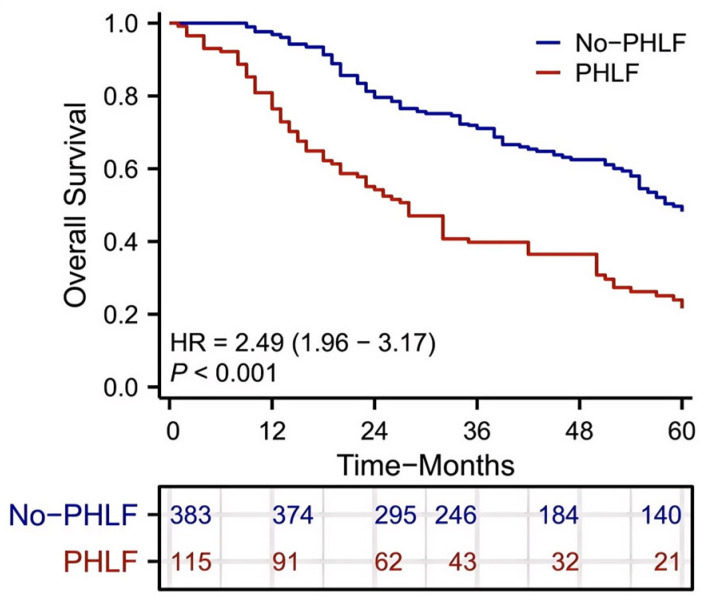
The Kaplan–Meier survival curve of overall survival in HCC patients with and without PHLF.

Kaplan–Meier analysis showed that patients who developed PHLF had significantly worse overall survival compared with those without PHLF (HR = 2.49, 95% CI: 1.96–3.17, *p* < 0.001). This analysis was not intended to evaluate model performance, but rather to illustrate the clinical significance of PHLF as a postoperative complication. The markedly worse survival observed in patients with PHLF highlights the importance of identifying high-risk patients before or during surgery. (Figure 2)

### 3.4. Comparison of Logistic Regression and Deep Learning Models

**Figure 3:**
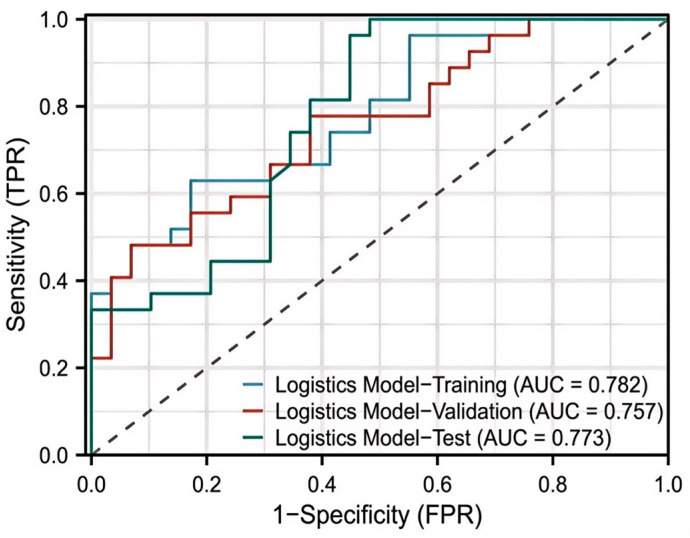
Performance of the Logistics Regression Model for ROC curve; the dashed diagonal line represents the line of no discrimination.

The logistic regression model was constructed using significant predictors identified from multivariate analysis. The model demonstrated an AUC of 0.782 in the training cohort, 0.757 in the validation cohort, and 0.773 in the test cohort (Figure 3).

**Figure 4:**
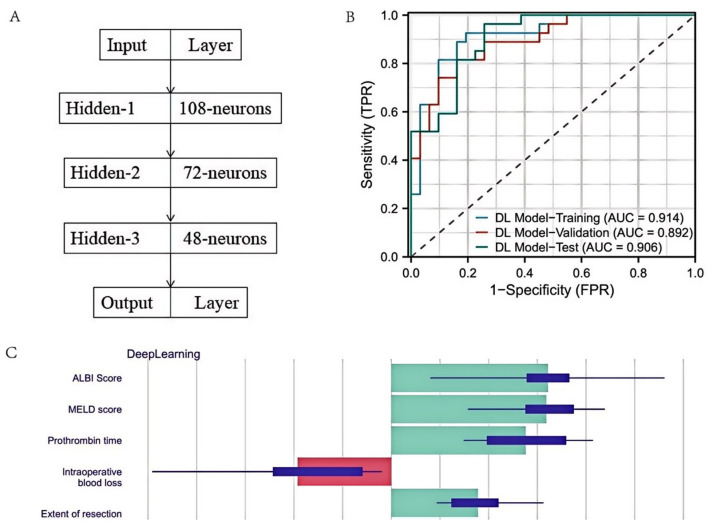
Performance of the Deep Learning Model. (**A**) represents the model architecture diagram, and (**B**) represents the ROC curve of the model on the training, validation and test sets; the dashed diagonal line represents the line of no discrimination. (**C**) represents the top five ranked SHAP scores of the deep learning model. Blue bars (right) indicate increased risk, whereas red bars (left) indicate decreased risk; bar length reflects the magnitude of feature contribution.

The deep learning model was developed using a fully connected neural network architecture designed to capture complex nonlinear relationships in the dataset. It featured an input layer for preoperative and intraoperative data, followed by three hidden layers (108, 72, and 48 neurons) with ReLU activation and dropout regularization (rate = 0.2). A sigmoid-activated output layer generated PHLF probability scores. The model was optimized with the Adam optimizer (learning rate = 0.001), used binary cross-entropy as its loss function, and incorporates early stopping to prevent overfitting. Figure 4A illustrates the model’s architecture.

After hyperparameter tuning, the deep learning model achieved an AUC of 0.914 in the training cohort, 0.892 in the validation cohort, and 0.906 in the test cohort (Figure 4B). Model interpretability was enhanced using SHapley Additive exPlanations (SHAP). The SHAP summary plot (Figure 4C) highlights that ALBI score, MELD score, and intraoperative blood loss had the highest contributions to PHLF prediction.

### 3.5. Performance of Multiple Models

The performance comparison between the models is summarized in Table 3. The deep learning model consistently outperformed logistic regression across all datasets, showing higher sensitivity, specificity, and F1-score. In the training, validation, and test cohorts, its AUCs were 0.914, 0.892, and 0.906, respectively, with corresponding F1-scores of 0.836, 0.814, and 0.825. Logistic regression had lower AUCs (0.782, 0.757, 0.773) and F1-scores (0.692, 0.683, 0.714). These results highlight the superior predictive capability of deep learning, supporting its potential as a more effective tool for PHLF risk assessment.

### 3.6. Calibration and Clinical Utility

Calibration curves and Brier scores for the deep learning and logistic regression models are presented in Supplementary Figure S1 and Supplementary Table S4. Decision curve analysis (Supplementary Figure S2) demonstrated the net benefit of the models across clinically relevant threshold probabilities, indicating potential clinical utility.

### 3.7. Sensitivity Analysis: Preoperative-Only Model

To evaluate preoperative applicability, we developed a preoperative-only model excluding intraoperative variables (e.g., operative time and blood loss). The preoperative-only model achieved an AUC of 0.821 in the test cohort, compared with an AUC of 0.906 for the perioperative deep learning model (Supplementary Table S5). Precision–recall (PR) curves were generated to evaluate model performance under class imbalance (Supplementary Figure S3). The deep learning model achieved higher average precision than logistic regression (MLP AP = 0.641 vs. LogReg AP = 0.578) and maintained higher precision across most recall ranges, indicating improved ability to identify patients at risk of PHLF.

## 4. Discussion

In this multicenter study, we demonstrated that deep learning models outperform traditional logistic regression in predicting post-hepatectomy liver failure (PHLF) in hepatocellular carcinoma (HCC) patients. The superior performance of the deep learning model is likely due to its ability to capture complex nonlinear interactions among clinical variables, which are often overlooked by conventional statistical approaches [21]. Logistic regression, while useful for understanding individual risk factors, has inherent limitations in modeling high-dimensional data and intricate relationships between preoperative liver function, intraoperative parameters, and postoperative outcomes. Our findings add to the increasing evidence supporting the use of artificial intelligence in potential utility for real-time surgical risk assessment. In our study, the logistic regression model showed moderate discrimination (test cohort AUC = 0.773), whereas the deep learning model achieved higher discrimination (test cohort AUC = 0.906).

Previous studies on PHLF prediction have primarily relied on clinical scoring systems or statistical models, with mixed results. The Model for End-Stage Liver Disease (MELD) score and the Albumin–Bilirubin (ALBI) score have been widely used for preoperative liver function assessment, but their predictive accuracy for PHLF remains suboptimal. For example, Wang et al. [22]. compared the Child-Pugh score, the MELD score, and the 15 min indocyanine green retention rate (ICG-R15) in evaluating liver function reserve, with results indicating that ICG-R15 was the most effective. The effectiveness of ALBI and MELD scores in predicting PHLF remains controversial. Zou et al. [23]. analyzed data from over 400 patients undergoing liver resection and compared different scoring models, finding that the ALBI score outperformed the Child-Pugh and MELD scores in predicting PHLF. Similarly, Tian et al. [24]. used logistic regression to identify factors associated with PHLF and demonstrated that combining the ALBI score with the FIB-4 index effectively predicted both PHLF and postoperative mortality in HCC patients undergoing hepatectomy. However, these studies did not assess the overall predictive accuracy of these scoring models, leaving uncertainty regarding the clinical utility of logistic regression in decision-making.

Currently, no deep learning models specifically designed for PHLF prediction have been developed. However, in recent years, the application of deep learning in postoperative risk prediction has gained increasing attention. Unlike logistic regression, deep neural networks can leverage hidden patterns in patient data, enabling more precise risk stratification. Several studies have demonstrated the advantages of machine learning for predicting postoperative complications. For example, Tashiro et al. [25] developed a machine learning model for predicting post-hepatectomy liver failure (PHLF) in 334 patients with liver cancer and reported that extreme gradient boosting achieved the best performance, with an AUROC of 0.863. Similarly, Wang et al. [26] constructed a multicenter machine learning model using LightGBM in a larger cohort of 875 patients with hepatocellular carcinoma, achieving an AUROC of 0.822 in the independent test cohort. Both studies also showed that machine learning models outperformed conventional liver function scores such as ALBI and FIB-4.

In the present study, we applied a deep learning framework for PHLF prediction and evaluated its performance using a multicenter dataset. The model integrates multiple clinical and surgical variables and is able to capture complex nonlinear relationships within the data. The resulting AUC values were 0.914, 0.892, and 0.906 in the training, validation, and test cohorts, respectively. Notably, the test cohort performance (AUC = 0.906) was comparable to or higher than that reported in previous machine learning studies, including Wang et al. (AUC = 0.822) and Tashiro et al. (AUC = 0.863). These findings further support the potential value of deep learning–based approaches for improving risk prediction of PHLF.

Several factors may explain these differences. First, our study used a predefined training–validation–test framework, allowing independent evaluation of model performance on unseen data. Second, the deep neural network architecture enables the model to capture complex nonlinear interactions among perioperative variables, which may not be fully captured by traditional statistical models or tree-based algorithms [27]. Finally, our study directly compared deep learning with conventional logistic regression using the same clinical variables, highlighting the potential incremental value of deep learning for perioperative risk prediction.

Feature importance analysis provided further insights into the key predictors of PHLF. SHapley Additive exPlanations (SHAP) values identified total bilirubin, albumin, prothrombin time, intraoperative blood loss, and extent of resection as the most influential variables contributing to model predictions. These findings are consistent with previous studies and further support the clinical relevance of these factors. For instance, Meng et al. [28] reported that intraoperative blood loss is an important determinant of postoperative hepatic function, while Lu et al. [29] highlighted the role of preoperative liver reserve indicators, such as albumin and prothrombin time. Taken together, these results suggest that combining indicators of preoperative liver function with intraoperative factors may provide a more comprehensive assessment of the risk of PHLF.

Accurate prediction of PHLF has important clinical implications. Patients identified as high-risk may benefit from targeted preoperative interventions, such as nutritional optimization, portal vein embolization to promote hypertrophy of the future liver remnant, or adjustments in surgical strategy to reduce intraoperative blood loss [2,8,30,31]. In addition, closer postoperative monitoring and early supportive management, including careful fluid management or prophylactic albumin supplementation, may help reduce the likelihood of severe liver failure. The integration of an artificial intelligence–based prediction tool into clinical practice could therefore support surgeons in patient selection and perioperative decision-making.

To further explore the potential clinical applicability before surgery, we also constructed a preoperative-only model using variables available prior to the operation. As expected, the predictive performance was lower than that of the perioperative model, indicating that intraoperative information contributes additional prognostic value. Nevertheless, the preoperative-only model still showed acceptable discrimination, suggesting that preliminary risk stratification may already be feasible in the preoperative setting.

Despite the promising results, our study has several limitations. First, the retrospective design may introduce selection bias and unmeasured confounding. Second, although data were collected from multiple centers, the evaluation was based on patient-level splitting of a pooled cohort and therefore constitutes internal multicenter validation rather than fully independent external validation. Third, all participating centers were located in China, which may limit generalizability to other populations and healthcare systems. Fourth, the inclusion of intraoperative variables supports perioperative risk updating but may limit purely preoperative use; we therefore added a preoperative-only sensitivity analysis. Finally, prospective validation and workflow-integrated implementation studies are needed before routine deployment. Future studies may further evaluate the comparative performance of deep learning with other machine learning algorithms in larger multicenter datasets.

## 5. Conclusions

This multicenter study demonstrates that deep learning improves predictive performance for post-hepatectomy liver failure compared with logistic regression in an internally validated multicenter cohort. By integrating preoperative variables and, when available, intraoperative parameters, the model may support perioperative risk stratification and surgical planning. Future research should prioritize prospective external validation and real-world implementation.

## Figures and Tables

**Table 1:** Baseline Characteristics of the Training, Validation, and Test Cohorts of HCC patients (n = 498).

Variables	Training Cohort (n = 349)	Validation Cohort (n = 75)	Test Cohort (n = 74)	*p*-Value
Age (years)	61 ± 7	60 ± 6	62 ± 8	0.421
Sex (Male, N (%))	237 (68.0%)	53 (70.7%)	51 (68.9%)	0.615
BMI (kg/m^2^)	26.4 ± 3.2	25.9 ± 3.1	26.1 ± 3.0	0.378
Diabetes (N (%))	94 (26.9%)	23 (30.7%)	21 (28.4%)	0.534
Hypertension (N (%))	133 (38.1%)	30 (40.0%)	29 (39.2%)	0.482
Preoperative Albumin (g/L)	39.8 [37.1, 42.2]	39.9 [36.9, 42.4]	39.5 [37.5, 42.0]	0.302
Preoperative Bilirubin (μmol/L)	15.4 [12.1–17.6]	14.9 [12.3–18.1]	15.1 [11.7–17.8]	0.423
Prothrombin Time (INR)	1.05 [1.00, 1.10]	1.05 [1.00, 1.11]	1.04 [1.00, 1.10]	0.364
MELD Score	10.4 ± 2.5	10.1 ± 2.3	10.3 ± 2.4	0.451
ALBI Score	−2.1 ± 0.4	−2.0 ± 0.4	−2.2 ± 0.4	0.498
ICG-R15 (%)	10.5 [6.4, 16.0]	11.0 [5.8, 16.5]	11.1 [6.2, 17.1]	0.513
Extent of Resection (Major, N (%))	175 (50.1%)	39 (52.0%)	38 (51.4%)	0.578
Intraoperative Blood Loss (mL)	260 [100–520]	280 [120–570]	250 [150–490]	0.618
Operative Time (minutes)	225 [200–260]	235 [217–278]	230 [196–285]	0.479
Postoperative Drainage (mL)	420 [200–550]	390 [180–570]	405 [195–590]	0.610
Preoperative AFP (ng/mL)	83.9 [6.4, 1600.8]	88.6 [5.1, 1019.5]	91.2 [5.17, 1804.2]	0.588
Child-Pugh Score (N (%))	280 (80.2%)	62 (82.7%)	60 (81.1%)	0.412
FIB-4 Index	3.2 ± 1.1	3.1 ± 1.0	3.3 ± 1.2	0.437
AST/ALT Ratio	1.1 [0.9–1.3]	1.0 [0.9–1.2]	1.1 [0.9–1.3]	0.402
Platelet Count (×10^9^/L)	167.5 [126.5, 220.2]	172.0 [129.2, 222.0]	161.5 [124.3, 214.0]	0.520
PHLF (N (%))	78 (22.3%)	18 (24.0%)	19 (25.7%)	0.248
PHLF Grade B (N (%))	48 (13.8%)	12 (16.0%)	13 (17.6%)	0.310
PHLF Grade C (N (%))	30 (8.6%)	6 (8.0%)	6 (8.1%)	0.450

The values in parentheses are percentages unless indicated otherwise. Continuous variables are presented as mean ± SD or median [IQR], as appropriate. Major hepatectomy was defined as resection of ≥3 Couinaud liver segments.

**Table 2:** Univariate and Multivariate Logistic Regression Analysis of Patients with Post-Hepatectomy Liver Failure (PHLF) in the Training Cohort.

Variables	Univariate OR (95% CI)	Univariate *p*-Value	Multivariate OR (95% CI)	Multivariate *p*-Value
Age (years)	1.25 (0.99–1.50)	0.079		
Sex (Male)	1.45 (1.10–1.82)	0.034	1.20 (0.96–1.45)	0.120
BMI (kg/m^2^)	0.98 (0.85–1.12)	0.451		
Diabetes	1.75 (1.30–2.20)	0.005	1.52 (1.20–1.95)	0.009
Hypertension	1.38 (1.10–1.70)	0.027	1.20 (0.95–1.45)	0.064
Preoperative Albumin (g/L)	0.87 (0.70–1.05)	0.178		
Preoperative Bilirubin (μmol/L)	1.60 (1.30–2.00)	0.002	1.45 (1.15–1.85)	0.006
Prothrombin Time (INR)	1.92 (1.50–2.40)	<0.001	1.78 (1.40–2.22)	0.001 #
MELD Score	1.68 (1.30–2.10)	0.004	1.55 (1.25–2.00)	0.008 #
ALBI Score	1.85 (1.40–2.30)	<0.001	1.72 (1.35–2.15)	0.002 #
ICG-R15 (%)	1.33 (1.10–1.65)	0.029	1.28 (1.05–1.55)	0.048
Extent of Resection (Major)	1.75 (1.35–2.25)	0.003	1.58 (1.25–2.08)	0.007 #
Intraoperative Blood Loss (mL)	1.42 (1.15–1.85)	0.011	1.38 (1.10–1.70)	0.015 #
Operative Time (minutes)	1.20 (0.95–1.55)	0.115		
Postoperative Drainage (mL)	1.12 (0.90–1.40)	0.189		
Preoperative AFP (ng/mL)	1.05 (0.88–1.28)	0.331		
Child-Pugh Score	1.82 (1.40–2.25)	<0.001	1.65 (1.30–2.10)	<0.001
FIB-4 Index	1.35 (1.12–1.72)	0.021	1.30 (1.05–1.60)	0.038
AST/ALT Ratio	1.15 (0.95–1.42)	0.127		
Platelet Count (×10^9^/L)	0.92 (0.75–1.12)	0.278		

# The final predictor variables were determined using the backward stepwise selection method and variance inflation factor (VIF). All variables had VIF values < 5, indicating no significant multicollinearity. Detailed VIF results are provided in Supplementary Table S3.

**Table 3:** Evaluation indicators for each model.

Metric	Logistic Regression (Training)	Logistic Regression (Validation)	Logistic Regression (Test)	Deep Learning (Training)	Deep Learning (Validation)	Deep Learning (Test)
AUC	0.782	0.757	0.773	0.914	0.892	0.906
Accuracy	0.722	0.711	0.735	0.856	0.834	0.843
Sensitivity	0.682	0.674	0.708	0.826	0.804	0.813
Specificity	0.754	0.733	0.762	0.880	0.866	0.873
Precision	0.703	0.693	0.721	0.846	0.825	0.838
F1-score	0.692	0.683	0.714	0.836	0.814	0.825
Brier score	0.150	0.148	0.140	0.120	0.125	0.133

Abbreviation: AUC: area under the curve.

## Data Availability

The data used in this study are not publicly available due to patient privacy and ethical restrictions. Data may be made available from the corresponding author upon reasonable request.

## Supplementary Materials

The following supporting information can be downloaded at: https://www.mdpi.com/article/10.3390/cancers18060926/s1, Supplementary Figure S1: Calibration plots; Supplementary Figure S2: Decision curve analysis; Supplementary Figure S3: Precision–recall curves; Supplementary Figure S4: Learning curves. Supplementary Table S1: Missingness and imputation strategy; Supplementary Table S2: Hyperparameter search space and final settings; Supplementary Table S3: VIF values for candidate predictors; Supplementary Table S4: Calibration metrics (including Brier score); Supplementary Table S5: Additional model performance details (including preoperative-only analysis); Supplementary Table S6: Model parameter count and implementation details; Supplementary Text S1: PASS sample-size estimation assumptions and results.

## Abbreviations

The following abbreviations are used in this manuscript:

AFP	Alpha-fetoprotein
ALB	Preoperative albumin
ALBI	Albumin–bilirubin score
ALT	Alanine aminotransferase
AST	Aspartate aminotransferase
AUC	Area under the receiver operating characteristic curve
BMI	Body mass index
FIB-4	Fibrosis-4 index
HCC	Hepatocellular carcinoma
IBL	Intraoperative blood loss
ICG-R15	Indocyanine green retention rate at 15 min
INR	International normalized ratio
IQR	Interquartile range
MELD	Model for End-Stage Liver Disease
OT	Operative time
PD	Postoperative drainage
PHLF	Post-hepatectomy liver failure
PR curve	Precision–recall curve
ROC	Receiver operating characteristic
SHAP	SHapley Additive exPlanations
