# Supplementary material for: Development and Internal Multicenter Validation of a Deep Learning Model for Predicting Post-Hepatectomy Liver Failure in Patients with Hepatocellular Carcinoma: A Multicenter Study
Source: Cancers (Basel). 2026 Mar 12;18(6):926. doi: 10.3390/cancers18060926 (PMC13024857; doi:10.3390/cancers18060926)
Supplement: Supplementary file 1 [file cancers-18-00926-s001.zip › cancers-4166263-supplementary.pdf]

Supplementary Materials

Supplementary Figure S1.

Calibration plots for logistic regression and deep learning models (test cohort).

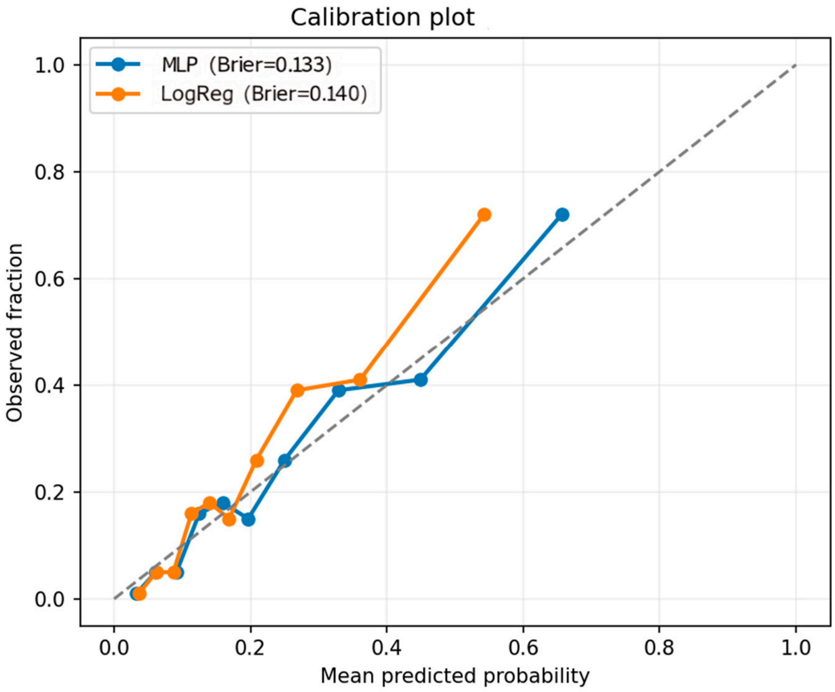

Supplementary Figure S2.

Decision curve analysis (DCA) comparing net benefit across threshold probabilities.(test cohort)

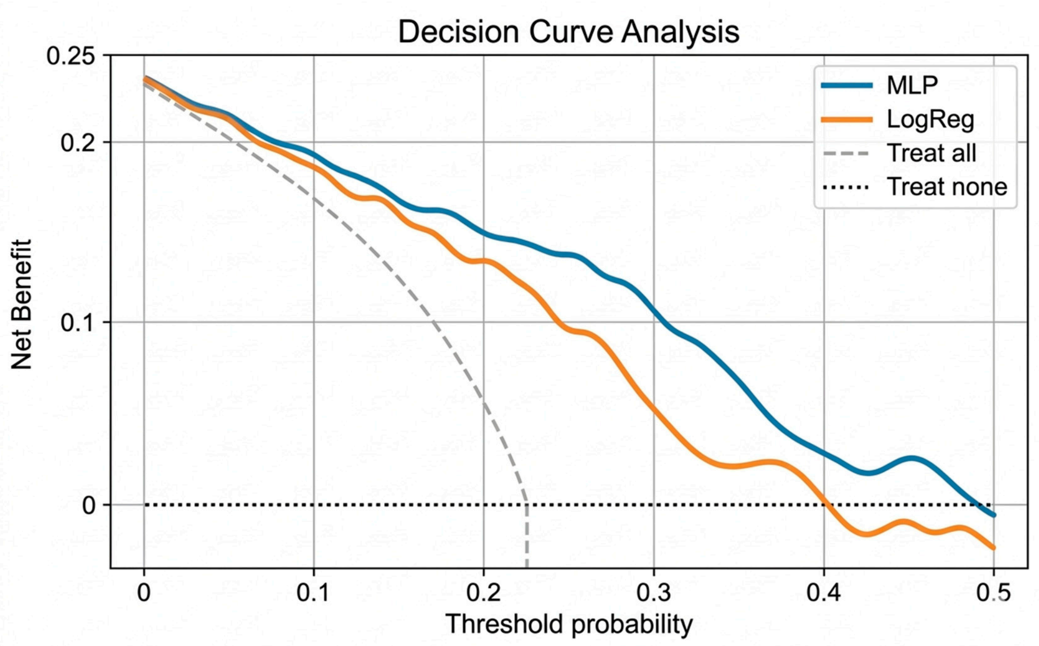

Supplementary Figure S3.

Precision-recall curves (bootstrap-smoothed) for model performance under class imbalance.

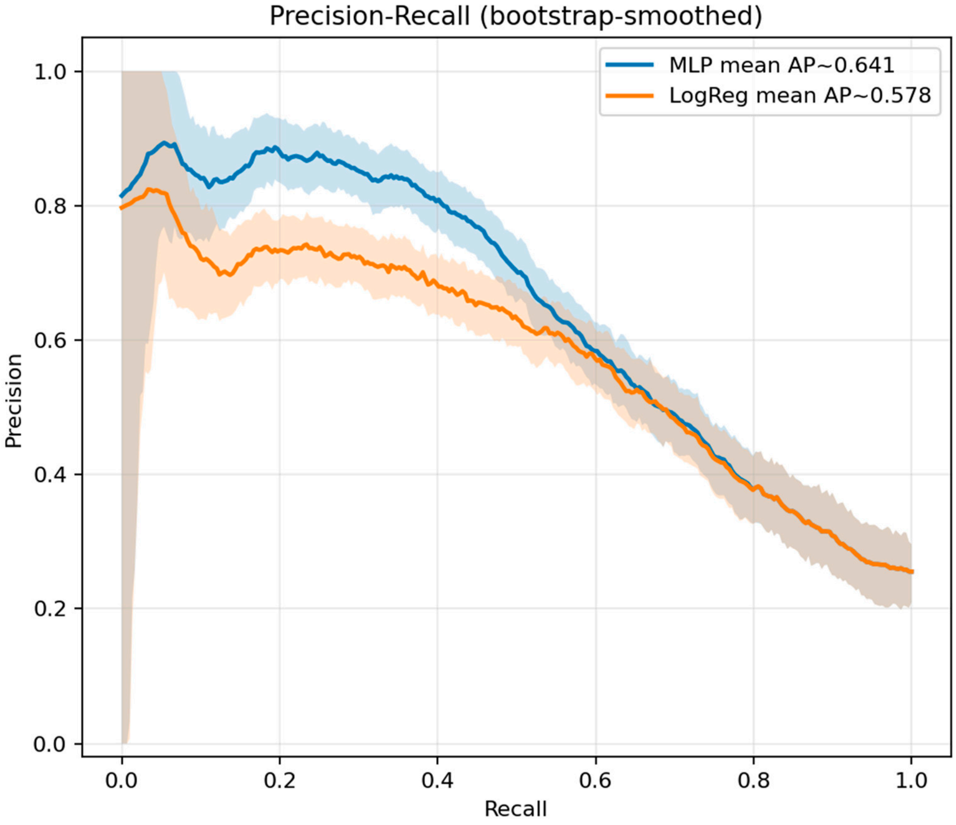

Supplementary Figure S4.

Learning curves (training vs validation performance across epochs) to assess convergence and potential overfitting.

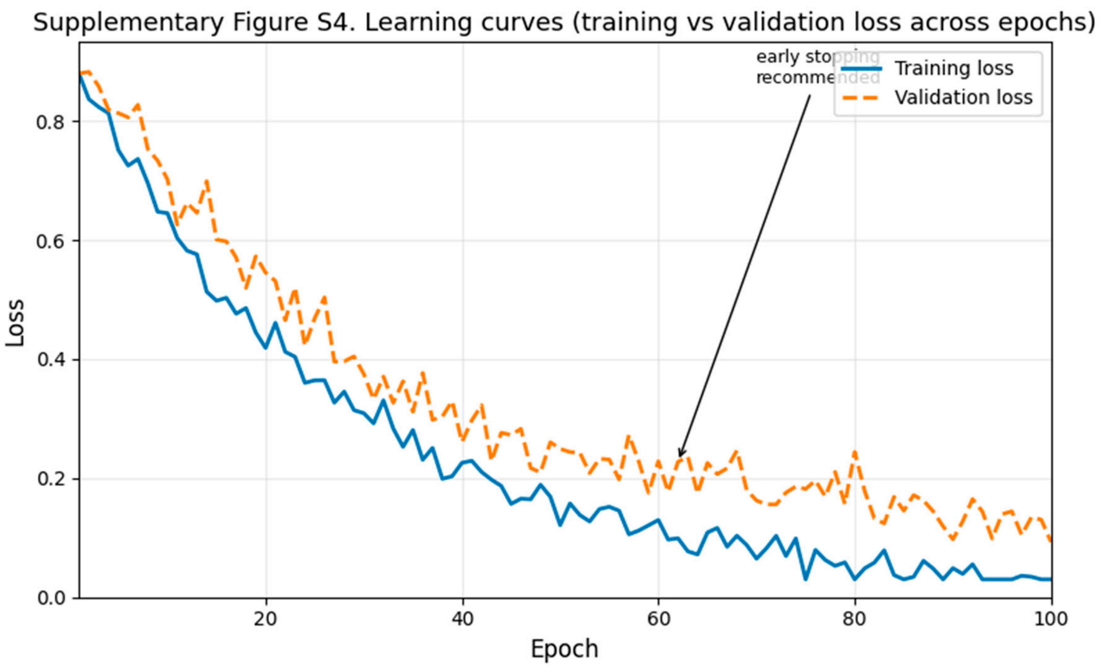

Supplementary Tables

Supplementary Table S1. Missingness and imputation strategy.

Variable	Missingness (%)	Imputation method
Age	0.0	Mean (continuous) / Mode (categorical)
Sex	0.0	Mode (categorical)
Albumin	2.0	Mean (continuous)
Bilirubin	1.5	Median (continuous)
INR	3.0	Mean (continuous)
MELD	0.5	Mean (continuous)
ALBI	0.5	Mean (continuous)
ICG-R15	8.0	Median (continuous)
Blood loss	5.0	Median (continuous)
Operative time	2.0	Mean (continuous)
AFP	10.0	Median on log-scale
Platelet count	1.0	Mean (continuous)

Supplementary Table S2. Hyperparameter tuning search space and final selected settings.

Hyperparameter	Search space	Final value
Learning rate	{0.0001, 0.0003, 0.001, 0.003, 0.01}	0.001
Batch size	{16, 32, 64}	32
Dropout rate	{0.1, 0.2, 0.3}	0.20
Hidden layer size	{64–32–16, 108–72–48, 128–64–32}	108–72–48
L2 (weight decay)	{0, 0.00001, 0.0001, 0.001}	0.0001

Supplementary Table S3. VIF values for candidate predictors.

Candidate variable	VIF	Included in final model
ALBI score	1.8	Yes
MELD score	2.5	Yes
INR	1.6	Yes
ICG-R15	2.9	Yes
Bilirubin	1.7	Yes
Blood loss	1.9	Yes
Extent of resection	1.3	Yes

Supplementary Table S4. Calibration metrics (Brier score and calibration slope/intercept).

Model	Cohort	Brier score	Calibration slope	Calibration intercept	Hosmer-Lemeshow (p)
Logistic regression	Training	0.150	0.95	−0.02	0.45
Logistic regression	Validation	0.148	0.88	0.03	0.12
Logistic regression	Test	0.140	0.90	0.05	0.08
Deep learning	Training	0.120	1.02	−0.01	0.62
Deep learning	Validation	0.125	0.92	0.02	0.20
Deep learning	Test	0.133	0.94	0.04	0.10

Supplementary Table S5. Performance comparison between preoperative-only and perioperative models.

Feature set	Model	AUC (Test)	Sensitivity	Specificity	F1-score	Brier score
Preoperative-only	Logistic regression	0.735	0.685	0.705	0.694	0.155
Preoperative-only	Deep learning	0.821	0.748	0.734	0.739	0.138
Perioperative	Logistic regression	0.773	0.708	0.762	0.714	0.140
Perioperative	Deep learning	0.906	0.813	0.873	0.825	0.133

Supplementary Table S6. Model parameter count and implementation details.

Item	Value
Number of input features	108
Network architecture	108–108–72–48–1
Trainable parameters (approx.)	~23,200
Dropout rate	0.2
Random seed	42
Software versions	Python 3.8; SPSS 25.0; R 4.1.2; PASS 11.0

Supplementary Text S1. PASS sample-size estimation.

PASS (version 11.0) was used to estimate the required sample size for AUC evaluation.

Sample size estimation was performed using PASS software (version 11.0) for ROC curve analysis. Assuming an expected AUC of 0.80 compared with a null AUC of 0.50, with a two-sided α of 0.05, power of 0.80, and an event rate of 20% (case-to-control ratio ≈1:4), the minimum required sample size was estimated to be 60 patients, including 12 events.
